# Comparison of preoperative and postoperative Lipid Profile changes in obese and morbidly obese patients after mini gastric bypass surgery

**DOI:** 10.12669/pjms.37.7.4123

**Published:** 2021

**Authors:** Kerim Guzel, Mustafa Ikizek

**Affiliations:** 1 Dr. Kerim Guzel, Assistant Professor, Department of General Surgery, Biruni University Faculty of Medicine, 34010 Topkapı, Istanbul, Turkey; 2 Mustafa Ikizek, Internal Medicine Clinic, Ankara, Turkey

**Keywords:** Diabetes mellitus, HDL, LDL, Mini-gastric bypass, Obesity, Total cholesterol, Triglyceride

## Abstract

**Background & Objective:**

Obesity has become a serious health problem that has become increasingly important in recent years. Since patients with high levels of obesity have dyslipidemia and an unbalanced lipid profile, they have a high risk of both diabetes mellitus and cardiovascular disease. This study aimed to evaluate the short (3 months) and long term (12 months) effects of mini-gastric bypass surgery from the current bariatric surgical techniques on the lipid profile.

**Methods:**

Of the patients undergoing Mini-gastric bypass operation between January 2016 to December 2018 at the General Surgery Clinic of Private Samsun Büyük Anadolu Hospital, demographic data and changes in lipid concentrations at 3 and 12 months were analyzed. Patients were grouped according to age, sex, body mass index (BMI), cardiologic risk groups, bypass lengths, and obesity classes. Total cholesterol, triglyceride, LDL-cholesterol, and HDL cholesterol values of the patients were examined at the time of admission to the outpatient clinic before the operation, at the postoperative third month and at the post-operative twelfth-month. Patients who did not go for a checkup during the one-year follow-up and whose data could not be reached or missing were excluded from the study.

**Results:**

There was no significant difference in terms of age, sex, and cardiovascular risk (p> 0.05). Although the HDL-C level was initially low (p <0.001), it significantly increased 12 months after surgical treatment (p <0.001). While serum concentrations of LDL cholesterol, total cholesterol, and triglycerides were high preoperatively, they significantly decreased at 12 months postoperatively (p <0.001). When compared with values in the 3rd- and 12th-month, there was a significant decrease in the class-3 obesity group but not in the class-2 obesity group. When serum HDL cholesterol concentrations were compared with preoperative baseline and postoperative 12th-month those, no statistically-significant difference was found in serum concentrations in the 3rd month, although there was a significant increase in both class 2 and 3 obesity groups.

**Conclusion:**

In patients undergoing mini-gastric bypass surgery, serum LDL cholesterol, total cholesterol, and triglyceride concentrations decreased in the 12th postoperative month, but serum HDL cholesterol concentrations increased.

## INTRODUCTION

Extremely obese individuals with insulin-resistant and dyslipidemia carry a high risk of Diabetes Mellitus (DM) and cardiovascular disease (CVD).^1^ Bariatric surgery is a surgical mode of treatment that causes long-term permanent weight loss and achieves the remission of comorbidities associated with excess body weight in many patients.^2^

The primary goal of different bariatric surgical procedures is to provide secondary benefits such as weight reduction in patients, increased insulin sensitivity and glycemic homeostasis, positive change in lipid profile, and a decrease in morbidity and mortality associated with cardiometabolic diseases.^3^ Although studies are showing that bariatric surgery causes long-term weight loss and changes lipid profile, limited information exists about the possible effects of this method on the lipid profile.^4^ We aimed here to extensively investigate the effect of mini-gastric bypass for bariatric surgery on short and long-term lipid profiles in obese and morbidly obese patients.

## METHODS

The study data was collected from morbidly obese patients whose body mass indexes were obese, i.e., 35<40 kg/m^2^ and 40 kg/m^2^ and over. These patients applied to Private Medicana International Samsun Hospital for overweight and obesity treatment between January 2016 and December 2018 and underwent mini-gastric bypass surgery. For this, informed consent was obtained from all patients before the study began. This was a retrospective study. Ethics committee approval was received from the Private Ethics Committee of Private Medicana International Samsun Hospital. The data were retrieved from the electronic registry system and patient files in the hospital where the operation was performed. Demographic data of the patients consisted of age, sex, body mass index, cardiologic risk groups (low/medium/high), bypass lengths, and obesity stages (stage 2 and 3). The BMI of obese (Grade-2) patients was 35<40 kg/m^2^ while that of the morbidly obese (Grade-3) patients was 40 kg/m^2^ and above. Total cholesterol, triglyceride, LDL-cholesterol and HDL cholesterol values of the patients were those at the time of admission to the outpatient clinic before the operation, within the postoperative third month, and at the postoperative twelfth-month follow-up.

### Data Extraction, Quality Assessment, and Follow-Up

Grade-2 obesity consisted of patients with a BMI of 35 <40 kg/m^2^, and Grade-3 obesity of those with a BMI of 40 kg/m^2^ and above. Total cholesterol (TC), triglyceride, LDL-cholesterol (LDL-C) and HDL cholesterol (HDL-C) values of the patients were those at the time of admission to the outpatient clinic before the operation, within the third postoperative month and at the post-operative twelfth-month follow-up. Patients over 18 years of age only were included in the study. Patients with Grade-1 obesity were excluded from the study. Patients who did not go for a checkup during follow-up and whose data could not be retrieved or is missing were excluded from the study.

### Operative Technique

**Fig.1:**
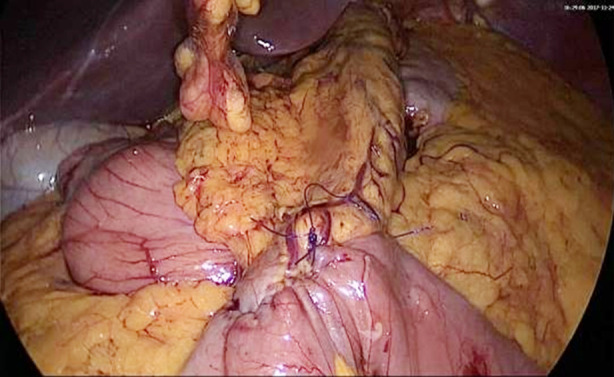
Mini-gastric Bypass. Gastric tube and gastrojejunostomy anastomosis

We operated on all our patients with the laparoscopic method by inserting five trocars. We used a fixable laparoscopic liver retractor. The stomach was sectioned with the help of an Endo-GIA at the level of the incisura angularis by protecting the Latarjet nerve. A 38 F calibration tube was inserted into the stomach with the help of an anesthetist. A narrow and long gastric tube with a capacity of 50-150 ml and a length of 15-18 cm was created by sectioning the stomach towards the angle of His in company with a tube using approximately 5 or 6 Endo-GIA along the small curvature in the vertical direction. Short gastric vessels were preserved. Most of the stapler lines were strengthened with a continuous suture of 3-0 polydioxanone. The distal jejunal loop ranging from 240 to 120 cm of the Treitz ligament was anastomosed antecolically to the gastric tube with the help of Endo-GIA. The stapler opening was sewn over with a continuous suture of 3-0 polydioxanone. Petersen Space of each patient was closed with a nonabsorbable continuous suture (Fig.1).

### Statistical Analysis

SPSS 25.0 (IBM Corporation, Armonk, New York, United States) and PAST3 (Hammer, Ø., Harper, D.A.T., Ryan, P.D. 2001, Paleontological statistics) were used to analyze the variables. The appropriateness of univariate data to normal distribution was tested by a Lilliefors-corrected Kolmogorov-Smirnov test and the variance homogeneity was by Levene test, while the appropriateness of multivariate data to the normal distribution was tested by Mardia (Dornik and Hansen omnibus) test and the variance homogeneity by Box-M test. Independent-Samples T-test and Bootstrap results were used to compare two independent groups, whereas the Mann-Whitney U test was used with the Monte Carlo simulation technique. Friedman’s Two-Way test was used to compare more than two repetitive measurements of dependent quantitative variables with each other, while Dunn’s Test was used for Post Hoc analysis. Quantitative variables were given as mean ± SD (Standard Deviation) (Minimum-Maximum) and median ± IQR (Interquartile Range) in the tables while the categorical variables were expressed as n (%). The variables were examined at a 95% confidence level, and the p-value less than 0.05 was considered statistically significant.

### Ethics approval

Ethics committee approval was received from the Private Ethics Committee of Private Medicana International Samsun Hospital, Ethical approval numbered 6307/2 07 dated 07 Oct 2019. The data were retrieved from the electronic registry system and patient files in the hospital where the operation was performed.

## RESULTS

A total of 146 patients underwent mini-gastric bypass surgery between January 2016 and December 2018. A total of 17 patients, including 12 patients with Grade-1 obesity and five patients with insufficient data were excluded from the study. A total of 129 patients, including 17 females and 8 males with Grade-2 obesity (n = 25) and 72 females and 32 males (n = 104) with Grade-3 obesity, were included in the study. The mean age of patients with Grade-2 obesity was 42.6 ± 12.1 years while that of patients with Grade-3 obesity was 45.3 ± 12.3 years. The mean BMI of patients with Grade-2 obesity was 38 ± 2 whereas that of patients with Grade-3 obesity was 47 ± 7.6. Of those with Grade-2 obesity, 13 carried low and 12 moderate cardiologic risks. On the other hand, of those with Grade-3 obesity, 44 had low, 59 moderate, and one high cardiologic risk. The median bypass length was 180 ± 25 cm in the Grade-2 obesity group while it was 200 cm in the Grade-3 obesity group. Bypass length was statistically significant and greater in patients with Grade-3 obesity (p <0.001). Although there existed a statistically significant difference between bypass lengths in obese and morbidly obese patients (p <0.05), no significant difference was observed in terms of age, gender and cardiovascular risk (p> 0.05) (Table-I).

**Table I:** Demographic data by obesity grade.

	*Obesity Grade*		*P*

	*II*	*III*	

	*(n=25)*	*(n=104)*	
Age, Mean ± SD (Min-Max)	42.6±12.1 (24 - 68)	45.3±12.3 (21 - 80)	0.332
BMI, Median ± IQR	38±2	47±7.6	<0.001

	*n(%)*	*n(%)*	

***Gender***			
Female	17 (68.0)	72 (69.2)	0.999 ^p^
Male	8 (32.0)	32 (30.8)	
***Cardiovascular Risk***			
Low	13 (52)	44 (42.7)	0.502 ^fe^
Moderate	12 (48)	59 (57.3)	
High exclude	0	1	

	*Median±IQR*	*Median±IQR*	

Bypass lengths	180±25	200±0	<0.001 ^u^

^t^Independent Samples T test (Bootstrap),

u Mann Whitney U test (Monte Carlo), p Pearson Chi-Sqaure Test (Exact), IQR: Interquartile Range, SD.: Standard deviaiton, Min.: Minimum, Max.: Maximum.

### Total Cholesterol

In the Grade-2 obesity group, there was a significant decrease in total cholesterol in the third and twelfth months compared to baseline (p <0.002, p <0.001). Similarly, in the Grade-3 obesity group, there was a significant decrease in total cholesterol in the third and twelfth months compared to baseline (p <0.001, p <0.001). Total cholesterol decreased significantly in the twelfth month compared to the third month (p <0.008) (Table-II).

**Table II:** Concentrations of serum lipid panels by obesity grade.

			Obesity Grade		^u^p value for between groups

			*II*	*III*	

			*(n=25)*	*(n=104)*	
COL		Baseline (A)	199±60	187±61	0.450
		3. Month (B)	156.5±38.5	162±48	0.722
		12. Month (C)	153±40.5	152±35	0.653
	Change			
		(B-A)	-25±30.5	-24±33	0.433
		(C-A)	-44.5±67.5	-36±47	0.611
		(C-B)	-10±32	-12±36	0.991
	^f^P Value for intra groups			
	Pairwise Comparation	A→B	0.002	<0.001	
		A→C	<0.001	<0.001	
		B→C	0.582	0.008	
TG		Baseline (A)	151±121	160±115	0.868
		3. Month (B)	112±34	117±53	0.298
		12. month (C)	105±42.5	100±49	0.306
	Change			
		(B-A)	-50.5±118.5	-35±98	0.383
		(C-A)	-47±142.5	-51±105	0.447
		(C-B)	-9.5±26.5	-11±32	0.659
	^f^P Value for intra groups		<0.001	<0.001	
	Pairwise Comparation	A→B	0.004	<0.001	
		A→C	<0.001	<0.001	
		B→C	0.182	0.005	
LDL		Baseline (A)	122.8±52.5	112.6±45.1	0.937
		3. month (B)	92.5±45.5	98±40.4	0.797
		12. month (C)	82±36.5	86.6±32	0.700
	Change			
		(B-A)	-17.6±28.7	-16±29.6	0.692
		(C-A)	-21.6±31.7	-19.2±32.4	0.898
		(C-B)	-7.5±12.1	-6±15.8	0.853
	^f^P Value for intra groups		<0.001	<0.001	
	Pairwise Comparation	A→B	0.018	<0.001	
		A→C	<0.001	<0.001	
		B→C	0.182	<0.001	
HDL		Bazal (A)	42±16	43±13.2	0.327
		3. Month (B)	44±8.5	42±13	0.201
		12. month (C)	52±14	49±18	0.216
	Change			
		(B-A)	4±8.2	0±11.1	0.320
		(C-A)	9±5.5	6±9	0.261
		(C-B)	6.5±6.5	6±8	0.673
	^f^P Value for intra groups		<0.001	<0.001	
	Pairwise Comparation	A→B	0.937	0.999	
		A→C	<0.001	<0.001	
		B→C	<0.001	<0.001	

u Mann Whitney U test (Monte Carlo),

f Friedman Test (Monte Carlo); Post Hoc Test: Dunn’s Test, IQR: Interquartile Range.

### Triglycerides

In the Grade-2 obesity group, there was a significant fall in triglyceride in the third and twelfth months compared to baseline (p<0.004, p <0.001). In a similar vein, in the class 3 obesity group, there was a significant decline in triglyceride in the third and twelfth months compared to baseline (p <0.001, p <0.001). Triglyceride declined significantly in the twelfth month compared to the third month (p <0.005) (Table-II).

### LDL-Cholesterol

In the Grade-2 obesity group, there was a significant fall in LDL-cholesterol in the third and twelfth months compared to baseline (p<0.018, p <0.001). Similarly, in the class 3 obesity group, there was a significant decline in LDL-cholesterol in the third and twelfth months compared to baseline (p <0.001, p <0.001). LDL-cholesterol was reduced significantly in the twelfth month compared to the third month (p <0.001) (Table-II).

### HDL-Cholesterol

There was a significant increase in HDL-cholesterol in the twelfth month compared to baseline, (p <0.001). There was a significant increase in HDL-cholesterol in the twelfth month compared to the third month (p<0.001). In the class 3 obesity group, although HDL cholesterol decreased in the third month compared to baseline, this was statistically insignificant (p>0.05). There was a significant increase in HDL cholesterol in the twelfth month compared to baseline (p <0.001). There was a significant increase in HDL cholesterol in the twelfth month compared to the third month (p <0.001) (Table-II).

## DISCUSSION

Today, obesity is one of the most common chronic metabolic diseases worldwide.^5^ Patients being under high risk for mortality are those with extremely high BMI, elderly, men, and multiple comorbidities or revision.^6^ Moderate rates of cardiac risk detected in the population with normal weight are within the range of 18 to 20%.^7^,^8^ Consistent with previous studies, our findings show that 48% of obese patients and 57% of morbidly obese patients are in the intermediate cardiologic risk group, and their rates are extremely high. However, our study indicates no significant difference was found between them in terms of the preoperative cardiologic risk when obese and morbid patients were compared with each other. The risk of developing any cardiac pathology in these patients can be explained by the presence of other factors that increase the risk rather than being caused by obesity alone.

The ultimate goal of bariatric surgical procedures is to achieve long-term permanent weight loss and the reduction of BMI to <35 kg/m^2^.^9^ Morbid obesity is defined as having a body mass index (BMI) of 40 kg/m^2^ and greater and shortens the survival period between 5 and 15 years.^10^ A study in patients with BMI> 50 kg/m2, BMI 40-50 kg/m^2^ and BMI <40 kg/m^2^ showed that permanent weight loss was greater in the groups with a high degree of obesity compared to the other groups, and that the rates of total body weight loss in the groups with higher BMI values were greater than groups with low BMI.^11^ The primary goal in patients we performed mini-gastric bypass on was to achieve the targeted weight loss. We classified the patients undergoing obesity surgery according to their degree of obesity and evaluated them for BMI, gender, age, bypass length, and CVD risks. We aimed to compare the changes in lipid profiles in the obese and morbidly obese group we thought to be demographically similar, except for the difference in body mass index. Having looked at the demographics of patients, we did not detect any significant difference in terms of age, sex, and cardiovascular risk in obese and morbidly obese patients (p> 0.05).

Of the patients that we performed mini gastric bypass surgery, the median bypass length was 180 ± 25 cm in the second-class obesity group and that in the third-class obesity group was 200 cm. The bypass length of the third-class obese patients was significantly higher than the others (p<0.05). This is because we made an effort to extend the bypass time slightly longer to increase weight loss more in morbid patients than in obese those.

According to 2018 data from the International Federation for the Surgery of Obesity and Metabolic Disorders (IFSO), the proportion of women to men among patients undergoing bariatric surgery is reported being significantly higher.^12^ Consistent with the literature, we found that the rate of female patients with class 2 obesity was 68% and that of female patients with class-3 obesity was 69%.

As evidenced by the studies, bariatric surgical procedures provide long-term effective weight loss in a way that will improve the obesity-related comorbidities.^13^ Some studies showed that bariatric surgery eliminates or improves cardiovascular risk factors such as diabetes, hypertension, and dyslipidemia.^14^ Reduction of lipase production with volumetric reduction following surgery leads to a significant decrease in the hydrolysis of triacylglycerols through a reduction in the released cholecystokinin and the absorption of free fatty acids.^15^ A long-term follow-up study on morbidly obese patients with Type-2 diabetes reported a 40% increase in triglyceride levels and a 20% increase in HDL cholesterol levels.^16^ It has been shown that malabsorptive bariatric surgery reduces sterol absorption and significantly lowers the total cholesterol and LDL concentrations by increasing cholesterol synthesis and catabolism.^17^ A recent study has shown that bariatric surgery affects the lipid profile, has an associated cardioprotective effect, and reduces significantly the total cholesterol, LDL, and triglyceride concentrations following an increase in HDL.^18^ As this study focuses mainly on the changes in lipid profile in obese and morbidly obese patients after a mini-gastric bypass, their other metabolic parameters were not evaluated.

The presence of a significant correlation between cardiovascular diseases and low serum HDL concentrations and high mortality rates has motivated researchers to develop medical strategies to increase low HDL concentrations.^19^ Among the recommended treatments to increase HDL concentrations in high-risk individuals with atherosclerotic diseases, there is increasing use of bariatric surgical methods that provide long-term permanent weight loss.^20^ Using the mini-gastric bypass surgery method for both obese and morbidly obese patients, we achieved a significant decrease in total cholesterol, triglyceride, and serum LDL cholesterol concentration at 3^rd^ month and an increase in serum HDL concentration at 12^th^ month.

A recent study evaluating the 15 months after bariatric surgery has found a significant increase in serum HDL concentration at the end of 15 months. The same study also reported a 2% to 3% reduction in the risk of cardiovascular disease for each 1 mg / dL increase in HDL concentrations.^21^ We detected an increase in HDL concentrations of patients with class 2 obesity at 3rd month compared to baseline, which was not statistically significant. Besides, we found an increase in HDL concentration at 12^th^ month compared to baseline, which was statistically significant. We observed a decrease in HDL concentrations of 3^rd^ class obese patients in the 3^rd^ month compared to baseline, which was statistically insignificant. However, we found an increase in HDL concentrations of the same patients in the 12^th^ month compared to baseline, which was statistically significant. Our results support both ideas in the literature. The differences in the data analysis by the obesity classes, that we classify according to BMI of the patients, reveal the importance of evaluating the obesity stages separately for future studies.

### Limitations of the study

The short follow-up period, failure to calculate the changes in body mass index, an omission of the patients with overweight and class 1 obesity, and different dietary habits with the ability to lead to changes in the accompanying lipid profile in some patients are limitations of this study.

## CONCLUSIONS

The present results suggest that the lipid profile improved after mini-gastric bypass surgery, the preoperative low HDL levels increased at the end of 12 months, and the preoperative high LDL, total cholesterol, and triglyceride levels decreased significantly at the end of three months and continued for 12 months. Based on our findings and literature, we consider that this may theoretically reduce cardiovascular risk and mortality rates.

### Author’s Contribution:

**KG:** Study design, data interpretation, final revision of the manuscript. **MI:** Statistical analysis, writing, improvement of English language. Both authors are responsible and accountable for the accuracy or integrity of the work.
